# The Dependence of Urinary Bladder Responses on Extracellular Calcium Varies Between Muscarinic, Histamine, 5-HT (Serotonin), Neurokinin, Prostaglandin, and Angiotensin Receptor Activation

**DOI:** 10.3389/fphys.2022.841181

**Published:** 2022-03-31

**Authors:** Charlotte Phelps, Russ Chess-Williams, Christian Moro

**Affiliations:** Centre for Urology Research, Faculty of Health Sciences and Medicine, Bond University, Robina, QLD, Australia

**Keywords:** bladder mucosa, G protein-coupled receptor (GCPR), l-type calcium channel, lamina propria, spontaneous activity, underactive bladder, muscularis mucosae

## Abstract

With many common bladder diseases arising due to abnormal contractions, a greater understanding of the receptor systems involved may aid the development of future treatments. The aim of this study was to identify any difference in the involvement of extracellular calcium (Ca^2+^) across prominent contractile-mediating receptors within cells lining the bladder. Strips of porcine urothelium and lamina propria were isolated from the urinary bladder dome and mounted in isolated tissue baths containing Krebs-bicarbonate solution, perfused with carbogen gas at 37°C. Tissue contractions, as well as changes to the frequency and amplitude of spontaneous activity were recorded after the addition of muscarinic, histamine, 5-hydroxytryptamine, neurokinin-A, prostaglandin E2, and angiotensin II receptor agonists in the absence and presence of 1 µM nifedipine or nominally zero Ca^2+^ solution. The absence of extracellular Ca^2+^ influx after immersion into nominally zero Ca^2+^ solution, or the addition of nifedipine, significantly inhibited the contractile responses (*p <* 0.05 for all) after stimulation with carbachol (1 µM), histamine (100 µM), 5-hydroxytryptamine (100 µM), neurokinin-A (300 nM), prostaglandin E2 (10 µM), and angiotensin II (100 nM). On average, Ca^2+^ influx from extracellular sources was responsible for between 20–50% of receptor-mediated contractions. This suggests that although the specific requirement of Ca^2+^ on contractile responses varies depending on the receptor, extracellular Ca^2+^ plays a key role in mediating G protein-coupled receptor contractions of the urothelium and lamina propria.

## 1 Introduction

While strong and sustained bladder contractions are vital for urinary voiding, during the filling stage abnormal and spontaneous contractions can result in bladder dysfunction. One common presentation is underactive bladder, characterized by a slow urinary stream, hesitancy and straining to void, with or without a feeling of incomplete bladder emptying, and sometimes with storage symptoms (Chapple et al., 2018). Normally, voiding commences when M3 muscarinic receptors in the smooth muscle of the bladder wall are stimulated by neuronally derived acetylcholine. However, recent research has identified a number of other receptor systems on cells within the bladder wall which may also modulate bladder contractions (Stromberga et al., 2019; Stromberga et al., 2020b). Of particular interest are those linked to G_q/11_ proteins, with their stimulation often resulting in strong tissue contractions. With many common bladder diseases arising as a result of abnormal contractions, an understanding of the potential receptor systems involved in this response is vital for the development of new and upcoming treatments for those affiliated with bladder dysfunctions.

A continuing interest has developed into the potential role of histamine (Stromberga et al., 2019), prostaglandins (Stromberga et al., 2020b), angiotensin (Lim et al., 2021), adrenergic (Moro et al., 2013), neurokinin (Grundy et al., 2018), muscarinic (Moro et al., 2011), and 5-hydroxytryptamine (Moro et al., 2016) receptors in influencing contractions of the urothelium and lamina propria (U&LP) layers. This layer has been observed to modulate overall bladder activity through the release of chemical mediators (Moro et al., 2011), as well as other pacemaking functions via the presence of the muscularis mucosae in the underlying connective tissue layer (Fry et al., 2007). The U&LP (also referred to as the bladder mucosa) may also play a key role in the mechanisms of action for current therapeutics (Moro et al., 2011), such as the parasympathomimetic bethanechol, which is one of the more commonly prescribed first-line treatments in the pharmaceutical management of underactive bladder (Kim, 2017). However, aside from this focus on the M3 muscarinic receptor, there is also a growing interest in combination therapies where, for example, administering both muscarinic agonists and alpha-adrenoceptor antagonists concurrently demonstrated greater success when compared to monotherapy in the management of underactive bladder (Yamanishi et al., 2004). This potential for alternative therapeutic options is important, as many patients who are administered muscarinic-acting pharmaceuticals in the management of bladder contractile disorders cease the regimen due to adverse side effects or lower than expected treatment outcomes (Yeowell et al., 2018). This is a growing area of research, with more work to be done regarding the potential benefits or harms from using parasympathomimetics in the management of underactive bladder (Moro et al., 2021).

Although the detrusor smooth muscle layer of the bladder has been the traditional target for research and therapeutic development, in recent years the importance of the U&LP in the maintenance of normal bladder function has been highlighted. The bladder generates spontaneous contractions, which can be altered in cases such as outlet obstruction or nerve injury (Kushida and Fry, 2016). The role of this spontaneous activity in the intact bladder is unclear (Fry and McCloskey, 2019), but may be mediated by signals originating in the U&LP, suggesting mechanisms where this tissue might underlie bladder contractile disorders (Fry and Vahabi, 2016). Calcium (Ca^2+^) and membrane potential transients commence in the lamina propria and spread towards the detrusor. In addition, the U&LP can release mediators, such as acetylcholine during stretching (Moro et al., 2011), which can induce increased spontaneous contractions in the detrusor (Kanai, 2007). A number of cells within the tissue may induce this spontaneous activity, as well as generate spontaneous Ca^2+^ transients, such as myofibroblasts (Sui et al., 2008), pericytes (Lee et al., 2016; Hashitani et al., 2018), interstitial-like cells (Fry and McCloskey, 2019) or muscularis mucosae (Heppner et al., 2011; Kushida and Fry, 2016). These cells remain highly sensitive to Ca^2+^, and there is value in identifying sources of Ca^2+^ influx into the tissue from extracellular fluids. Of note, however, is the potential for species differences in the generation and activity of phasic contractions. For example, the spontaneous activity of the bladder urothelium and lamina propria in the pig appears to predominantly arise from the muscularis mucosae, although there is evidence that this is not the case across all species (Mitsui et al., 2020).

With stimulation of the G protein-coupled receptors (GPCRs) resulting in contractions of the urinary bladder U&LP, identifying any common mechanisms of action between them is important. One primary function of the GPCRs in the urinary bladder may be the modulation of Ca^2+^ channels in the cell membranes, accommodating an influx of Ca^2+^ from extracellular fluids, and mediating a variety of physiological responses, from bladder contractions to increased pacemaker activity (Wuest et al., 2007). This study aims to identify similarities in extracellular Ca^2+^ requirements between muscarinic, histamine, 5-hydroxytryptamine (5-HT), neurokinin-A (NKA), prostaglandin E2 (PGE2), and angiotensin II (ATII) receptors for mediating contractile activity of the urinary bladder U&LP.

## 2 Materials and Methods

### 2.1 Tissue Collection

Urinary bladders from Large White-Landrace-Duroc pigs (6 months old, 80 kg live weight) were used as the tissue in this study. All bladders were obtained from the local abattoir after slaughter for the routine commercial provision of food with no animals bred, harmed, culled, interfered, or interacted with as part of this research project. As such, animal ethics approval was not required (Business Queensland, 2015). Only female bladders were used in this study. After collection from the abattoir, tissues were transported in a portable cooler in cold Krebs-Henseleit bicarbonate solution (NaCl 118.4 mM, NaHCO_3_ 24.9 mM, D-glucose 11.7 mM, KCl 4.6 mM, MgSO_4_ 2.41 mM, CaCl_2_ 1.9 mM, and KH_2_PO_4_ 1.18 mM) maintained at 4 °C to the University research facilities and used within 3 hours of the animal’s slaughter.

### 2.2 Tissue Preparation

A single 4 cm strip was taken longitudinally from the bladder wall at the midpoint between the trigone and the bladder apex. Tissue strips were continually washed with a cold Krebs-Henseleit bicarbonate solution during the preparation and dissection stage. The white-coloured detrusor smooth muscle was dissected away from the pink-coloured urothelium and lamina propria using fine scissors. Histological assessment is known to effectively separate the two layers (Moro et al., 2012). After dissection and preparation, this single strip was cut in the middle, resulting in 2 × 2 cm strips which were mounted and suspended in 10 ml isolated tissue baths (Labglass, Brisbane, Australia) containing warmed Krebs solution at 37°C and perfused with carbogen gas (95% oxygen and 5% carbon dioxide). A maximum of two 4 cm strips were taken from each unique porcine bladder across the experiments. Throughout this manuscript, *n* values quoted are from paired tissue strips, and as such, the number of animals (N) used can be calculated using n÷2 . After mounting, tissue tension was manually adjusted to 2 g using each transducer positioner’s fine adjustment knob. Each bath was washed with warmed Krebs a total of three times prior to the start of experimentation. At the conclusion of each experiment, tissue weight was measured on a scale to an accuracy of 0.001 g. The mean weight of porcine U&LP tissues was 0.22 ± 0.01 g (*n* = 208).

### 2.3 Pharmaceutical Agents

Krebs-Henseleit bicarbonate solution ingredients were obtained from Sigma-Aldrich (Missouri, U.S.). Carbamylcholine chloride (carbachol) and histamine dihydrochloride (histamine) were obtained from Sigma-Aldrich (Missouri, U.S.), nifedipine and NKA were from Tocris Bioscience (Bristol, U.K.), 5-HT was from Toronto Research Centre (Toronto, CA), and ATII and PGE2 were obtained from Cayman Chemicals (Michigan, U.S.). Nifedipine and PGE2 were dissolved in 100% ethanol, while all other pharmaceutical agents were soluble in distilled water. Concentrations chosen for the agonists and antagonists were selected based on their selectivity at each receptor and consistent with concentrations used in previous studies. In each case, the agonist concentration used induced a submaximal contraction (with an aim to achieve around 80% of peak receptor-induced contraction).

### 2.4 Measurements and Data Collection

In the nifedipine studies, a vehicle control of 100% ethanol was added to control tissues or nifedipine (1 µM) was added to the experimental tissues and left to equilibrate for 30 min. Nifedipine was kept in darkness until the final application in the organ bath and experiments were concluded within 30 min to ensure no adverse light impacts. In the Ca^2+^-free studies, control tissues were washed with Krebs solution as normal, whereas the experimental tissues were washed three times with a nominally Ca^2+^-free Krebs solution. This also ensured that any excess Ca^2+^ on the tissue, or Ca^2+^ leaking out from intracellular sources, was cleared from the bath. Tissues were then left to equilibrate for 3 min in nominally zero Ca^2+^ solution before agonists were added. A single dose of a select GPCR agonist was added to both the control and experimental tissues after equilibration. Tension, frequency, and amplitude of spontaneous contractions were measured with an isometric force transducer (MCT050/D, ADInstruments, Castle Hill, Australia) and recorded on a Powerlab system using Labchart v7 software (ADInstruments). Although a threshold was not applied, in all cases each spontaneous contraction exceeded 0.4 g.

### 2.5 Statistical Analysis

Changes in tension and amplitude were measured in grams (g), where amplitude was measured from the lowest point of spontaneous phasic contraction to peak. Frequency was expressed as the number of spontaneous phasic contractions per minute (cpm). All data was analysed using GraphPad Prism version 9 (San Diego, CA), and results were presented as mean ± standard error of the mean (SEM). A paired *t*-test was used to analyse tissue responses before and after the addition of agonist. A paired Student’s two-tailed *t*-test was used to analyse the significance of results when comparing tissues with direct controls, as per previous studies (Stromberga et al., 2020a). A one-way ANOVA with Tukey post-test was also undertaken to compare the means where more than two variables were assessed. For all statistical analysis, *p* < 0.05 was considered statistically significant.

### 2.6 Preliminary Results Related to the Omission of Extracellular Ca^2+^

There is variation in the literature surrounding methods to remove extracellular Ca^2+^ from an isolated tissue bath. Firstly, this may involve the omission of calcium chloride (CaCl) from Krebs-Henseleit bicarbonate solution (Poyser, 1984). Secondly, the addition of Ca^2+^ chelating agents ethylenediaminetetraacetic acid (EDTA, 1–5 mM) (Maggi et al., 1989) or glycoletherdiaminetetraacetic acid (EGTA, 0.5 mM) solution (Yoshimura and Yamaguchi, 1997) have been proposed. However, EGTA may have impacts on other ions (Wheeler and Weiss, 1979) or other mechanisms within the tissue (Poyser, 1984), as well as potentially destabilise cell membranes, leading to increased Ca^2+^ permeability (Guan et al., 1988). To assess this feasibility of using Ca^2+^ chelators, we observed that compared to the simple omission of CaCl, the addition of 1 mM EDTA or 0.5 mM EGTA had no effect on contractile responses to our agonists (e.g., carbachol 1 µM). Thirdly, in some studies, the removal of Ca^2+^ appeared to result in a hypovolemic solution, and in order to rectify this, the concentration of other ions was altered. In our pilot studies, when incorporating additional Mg^2+^ to substitute for omitted Ca^2+^, there was no significant effect on contractions (*n* = 8). As such, no additional magnesium was added to replace the omission of the Ca^2+^ divalent cation. Fourthly, there is the potential for Ca^2+^ to enter the Ca^2+^-free bath solution from the cytoplasm, or via the activation of other cellular pumps/exchangers. To minimise the impact of this, after mounting in the bath, tissues were washed three times over 5 min in warmed Ca^2+^-free Krebs. As a final check, before and after contractions, the extracellular buffer was collected and checked with spectrophotometric assessments (Pacific Laboratory Products, Victoria, Australia), with no Ca^2+^ detected on this assay. This provided confidence in a nominally zero Ca^2+^ solution. As there were no observable or significant impacts to any contractions from adjusting for the methods listed above, the decision was made to henceforth solely remove Ca^2+^ from the Krebs solution and not alter anything else, as this was the path of least manipulation.

## 3 Results

### 3.1 Influence of GCPR Agonists in U&LP

In the absence of stimulation from any agonist, U&LP tissue strips developed spontaneous phasic contractions at a frequency of 3.65 ± 0.08 cpm with an amplitude of 0.73 ± 0.04 g (*n* = 104). When receptor agonists carbachol (1 µM), histamine (100 µM), 5-HT (100 µM), NKA (300 nM), PGE2 (10 µM), and ATII (100 nM) were added to the tissues, U&LP baseline tension increased significantly for all activated receptors (*p* < 0.001, Table 1). In addition, the frequency of spontaneous phasic contractions increased for carbachol (*p* < 0.001), histamine (*p* < 0.05), 5-HT (*p* < 0.01), and ATII (*p* < 0.05), but not NKA or PGE2. Amplitude was reduced by all the agonists, but the changes were statistically significant for only carbachol (*p* < 0.05), 5-HT (*p* < 0.001), and ATII (*p* < 0.01).

**TABLE 1:** Summary of U&LP tissue spontaneous phasic activity. Tension, frequency, and amplitude recorded in response to receptor agonists. Data presented as mean ± SEM.

Agonist	Conc	Δ Tension (g)	Δ Frequency (cpm)	Δ Amplitude (g)	*n*
Carbachol	1 µM	3.77 ± 0.31***	1.15 ± 0.20***	−0.27 ± 0.10*	22
Histamine	100 µM	1.54 ± 0.20***	0.64 ± 0.24*	−0.08 ± 0.07	16
5-HT	100 µM	6.23 ± 0.64***	2.15 ± 0.64**	−0.37 ± 0.09***	17
NKA	300 nM	2.50 ± 0.25***	0.11 ± 0.24	−0.12 ± 0.06	17
PGE2	10 µM	2.43 ± 0.22***	−0.05 ± 0.34	−0.03 ± 0.04	16
ATII	100 nM	1.50 ± 0.18***	0.20 ± 0.08*	−0.22 ± 0.06**	16

*p < 0.05, **p < 0.01, ***p < 0.001 (paired *t*-test).

### 3.2 Influence of Nifedipine on U&LP Contractions

When nifedipine (1 µM) or vehicle control (totaling 0.035% ethanol) were added to tissues at the start of the 30 min equilibration period, there were no immediate changes to baseline tension, frequency, or amplitude for any of the agonists.

#### 3.2.1 Effect of Nifedipine on Baseline Tensions

**FIGURE 1:**
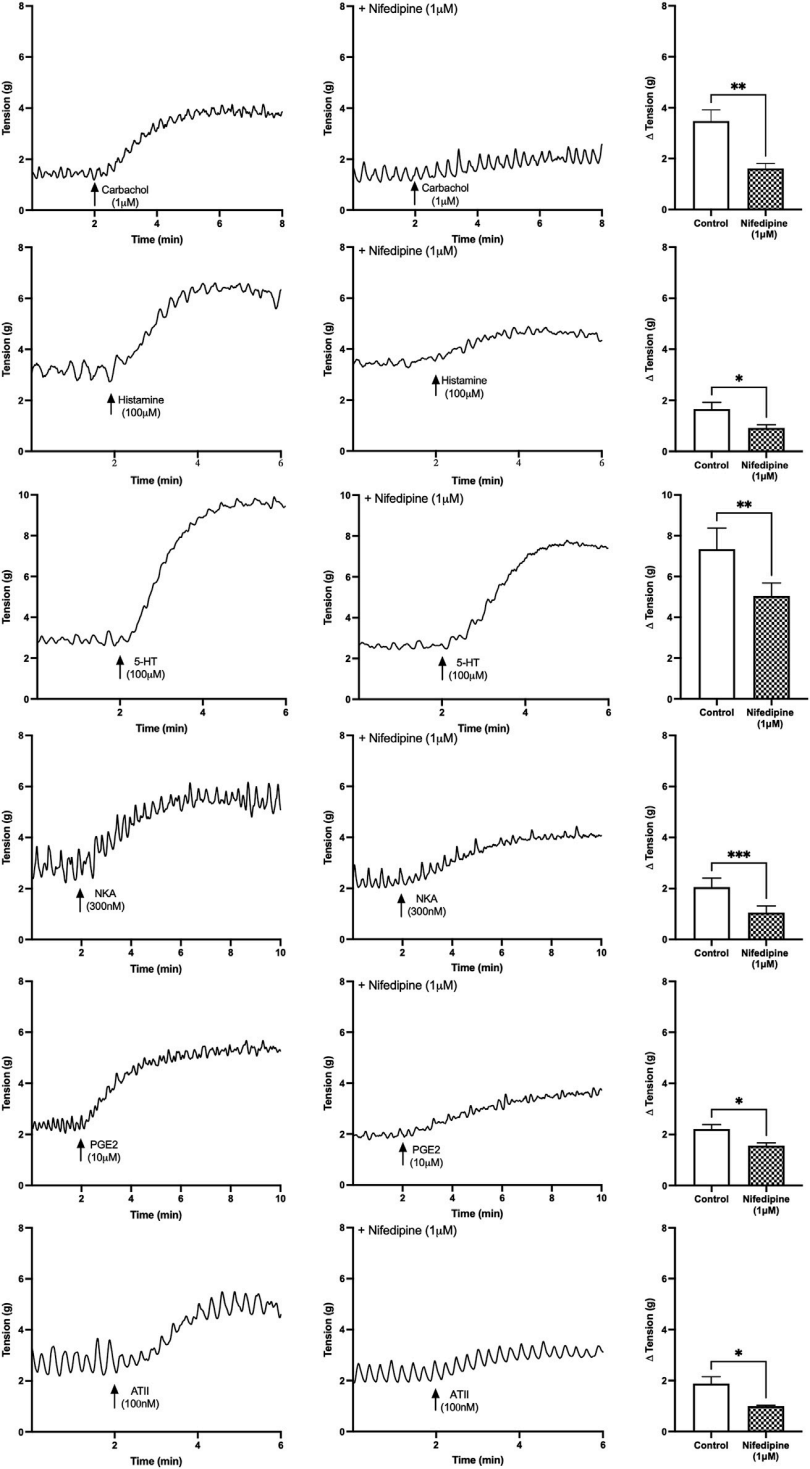
U&LP baseline tension responses to receptor agonists carbachol (1 µM), histamine (100 µM), 5-HT (100 µM), NKA (300 nM), PGE2 (10 µM), and ATII (100 nM) in the absence of (left) and the presence of (right) nifedipine (1 µM). *p < 0.05, **p < 0.01, ***p < 0.001 (paired Student’s two-tailed *t*-test).

After activation of the muscarinic, histamine, 5-HT, NKA, PGE2 and ATII receptors, the baseline tension of the U&LP tissue increased, and the increases were similar for all the agonists (Figure 1). In the presence of nifedipine (1 µM), the contractions were inhibited as follows (paired Student’s two-tailed *t*-tests): carbachol by 54% (1µM, *n* = 11, *p* < 0.01); histamine by 45% (100µM, *n* = 8, *p* < 0.05); 5-HT by 28% (100µM, *n* = 8, *p* < 0.01); NKA by 49% (300nM, *n* = 8, *p* < 0.001); PGE2 by 29% (10µM, *n* = 8, *p* < 0.05); and ATII by 47% (100nM, *n* = 8, *p* < 0.05). The impact of nifedipine was relatively consistent after the activation of each agonist, with no significant differences found between any of the responses (*p* = NSD, ANOVA with Tukey post-test for this final statistical assessment only).

### 3.2.2 Effect of Nifedipine on Frequency and Amplitude of Phasic Contractions

The frequencies and amplitudes of spontaneous phasic contractions produced by the U&LP tissues were investigated in the absence and presence of nifedipine for each of the receptor agonists (Table 2). No significant differences in the contractile frequency or amplitude were observed between the presence and absence of nifedipine (1 µM) for responses to carbachol, histamine, 5-HT, or NKA. Contractions for PGE2 (10µM, *n* = 8, *p* < 0.01) and ATII (100nM, *n* = 8, *p* < 0.05) exhibited a reduction in the amplitude of spontaneous phasic contractions in the presence of nifedipine, and when compared to the response in the absence of nifedipine, was a statistically significant difference.

**TABLE 2:** U&LP change in frequency and amplitude responses to receptor agonists in the absence (control) and presence of nifedipine (1 µM). There were no significant differences between the average frequency changes between the absence and presence of nifedipine for any of the agonists. Data presented as mean ± SEM.

	Δ Frequency (cpm)			
**Agonist**	**Conc**	**Control**	**+ Nifedipine**	***p*-value**	***n***
Carbachol	1 µM	0.92 ± 0.17***	1.20 ± 0.44*	0.58	11
Histamine	100 µM	0.76 ± 0.28*	0.83 ± 0.33*	0.88	8
5-HT	100 µM	2.58 ± 0.95*	3.67 ± 1.66	0.26	8
NKA	300 nM	0.44 ± 0.40	0.50 ± 0.59	0.91	8
PGE2	10 µM	-0.23 ± 0.38	-0.41 ± 0.41	0.78	8
ATII	100 nM	0.06 ± 0.17	0.24 ± 0.18	0.45	8
**Δ Amplitude (g)**					
Carbachol	1 µM	−0.51 ± 0.17*	−0.18 ± 0.06*	0.07	11
Histamine	100 µM	−0.03 ± 0.05	−0.07 ± 0.04	0.54	8
5-HT	100 µM	−0.25 ± 0.10*	−0.28 ± 0.08**	0.70	9
NKA	300 nM	−0.23 ± 0.08*	−0.18 ± 0.07*	0.48	8
PGE2	10 µM	−0.04 ± 0.04	−0.16 ± 0.03***	0.01**	8
ATII	100 nM	−0.32 ± 0.08**	−0.16 ± 0.06**	0.05*	8

*p < 0.05, **p < 0.01, ***p < 0.001 (paired *t*-test) after the addition of each agonist (listed to the left) in the absence and presence of nifedipine (1 µM). *p*-values (paired Student’s two-tailed *t*-test) in the right column denote the differences between the responses to agonist in the absence of and the responses to agonist in the presence of nifedipine.

### 3.3 Influence of Nominally Zero Ca^2+^ Solution on U&LP Contractions

#### 3.3.1 Effect of Nominally Zero Ca^2+^ Solution on Baseline Tensions

**FIGURE 2:**
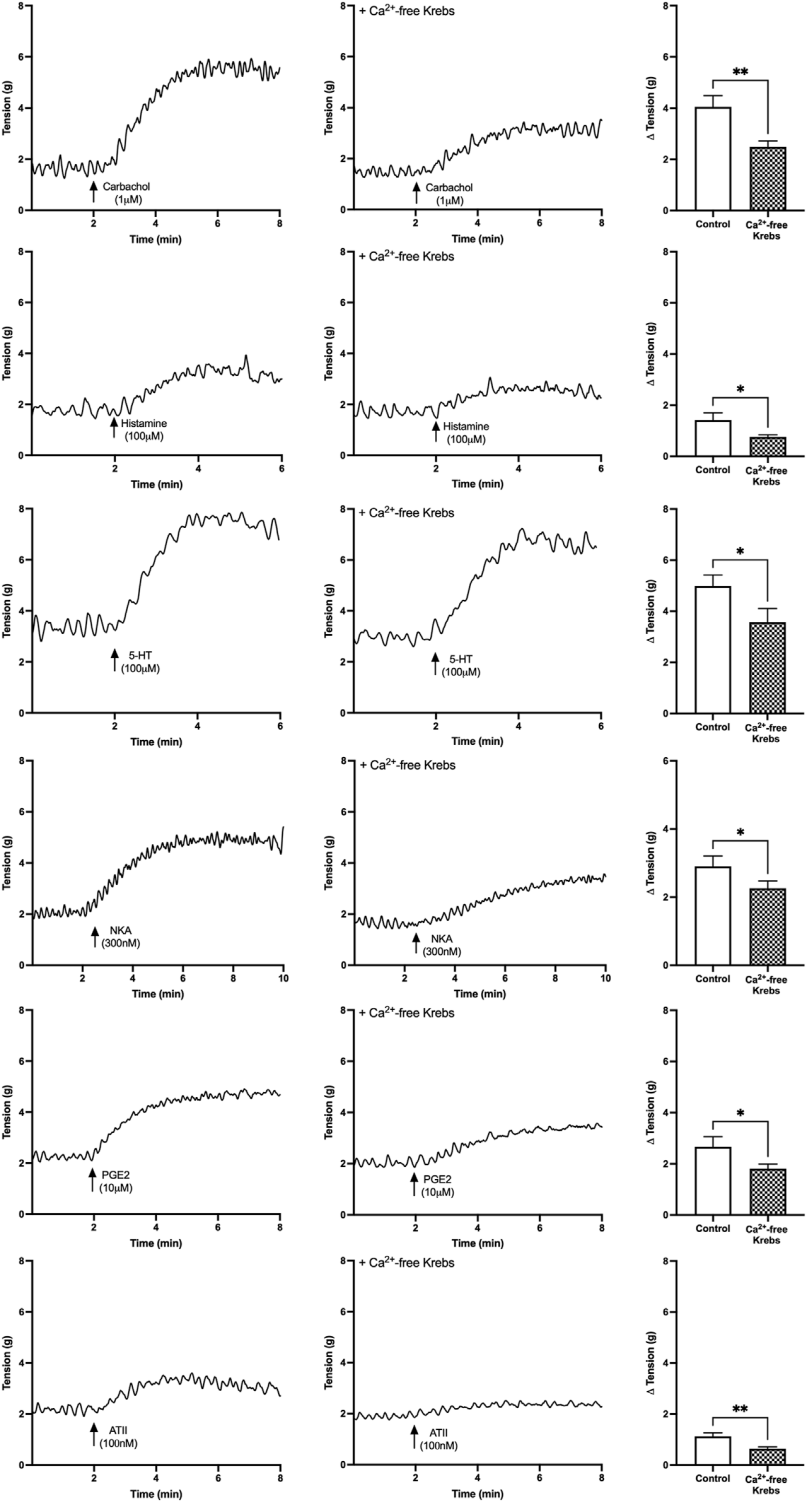
U&LP baseline tension responses to receptor agonists carbachol (1 µM), histamine (100 µM), 5-HT (100 µM), NKA (300 nM), PGE2 (10 µM), and ATII (100 nM) as controls in the normal Krebs (left) and in nominally zero Ca^2+^ solution (right). *p < 0.05, **p < 0.01, ***p < 0.001 (paired Student’s two-tailed *t*-test).

U&LP contractions for all receptor agonists in the presence of nominally zero Ca^2+^ solution were significantly inhibited compared to the control group in normal Krebs solution. In the absence of any extracellular Ca^2+^ sources, the contractions of the U&LP were impaired (paired Student’s two-tailed *t*-test, Figure 2). Contractions were inhibited as follows: carbachol by 39% (1µM, *n* = 11, *p* < 0.01); histamine by 46% (100 µM, *n* = 8, *p* < 0.05); 5-HT by 28% (100 µM, *n* = 8, *p* < 0.05); NKA by 22% (300 nM, *n* = 9, *p* < 0.05); PGE2 by 32% (10 µM, *n* = 8, *p* < 0.05); and ATII by 43% (100 nM, *n* = 8, *p* < 0.01). Across all receptors, there were no significant differences between the averaged responses (*p* = NSD, ANOVA with Tukey post-test).

#### 3.3.2 Effect of Nominally Zero Ca^2+^ Solution on Frequency and Amplitude of Phasic Contractions

Tissues exposed to a Ca^2+^-free extracellular environment had no alterations to their frequency or amplitude of spontaneous phasic contractions during responses to receptor agonists carbachol (1 µM), histamine (100 µM), 5-HT (100 µM), NKA (300 nM) or PGE2 (10 µM). Although the activation of all receptors induced increases to baseline tensions, only ATII (100 nM) also resulted in the significant decrease in the frequency of spontaneous phasic contractions in the presence of nominally zero Ca^2+^ solution (Table 3).

**TABLE 3:** U&LP change in frequency and amplitude responses to receptor agonists in the absence (control) and presence of nominally zero Ca^2+^ solution. No significant differences between the average amplitude changes between the absence and presence of nominally zero Ca^2+^ solution for any of the agonists. Data presented as mean ± SEM.

	Δ Frequency (cpm)			
**Agonist**	**Conc**	**Control**	**+ Ca^2+^-free Krebs**	***p*-value**	***n***
Carbachol	1 µM	1.38 ± 0.36**	1.88 ± 0.84*	0.48	11
Histamine	100 µM	0.74 ± 0.27*	0.93 ± 0.70	0.52	6
5-HT	100 µM	2.44 ± 0.77*	2.68 ± 1.31	0.83	8
NKA	300 nM	0.01 ± 0.24	1.93 ± 0.47	0.09	9
PGE2	10 µM	0.14 ± 0.59	0.63 ± 0.45	0.49	8
ATII	100 nM	0.22 ± 0.09*	−0.32 ± 0.16	0.02*	8
**Δ Amplitude (g)**					
Carbachol	1 µM	−0.02 ± 0.05	0.02 ± 0.05	0.58	11
Histamine	100 µM	−0.13 ± 0.12	−0.19 ± 0.07*	0.71	8
5-HT	100 µM	−0.51 ± 0.14**	−0.22 ± 0.22	0.17	8
NKA	300 nM	−0.01 ± 0.07	−0.05 ± 0.05	0.63	9
PGE2	10 µM	−0.02 ± 0.08	−0.08 ± 0.08	0.66	8
ATII	100 nM	−0.12 ± 0.08	0.04 ± 0.11	0.32	8

*p < 0.05, **p < 0.01 (paired *t*-test) after the addition of each agonist (listed to the left) in the absence and presence of Ca^2+^-free Krebs. *p*-values (paired Student’s two-tailed *t*-test) in the right column denote the differences between the responses to agonist in the absence of and the responses to agonist in the presence of nominally zero Ca^2+^ solution.

### 3.4 Overall Impact of Extracellular Ca^2+^ on Baseline Tension Contractions

In this study, two different methods were applied to assess the impact of extracellular Ca^2+^: the immersion of the tissue in nominally zero Ca^2+^ solution; or through Ca^2+^ channel antagonism with nifedipine (1 µM). Both methods significantly inhibited contractile activity changes in response to the assessed agonists. Overall, when looking at the impact of either nifedipine or nominally zero Ca^2+^ solution, there were no significant differences (Student’s two-tailed unpaired *t-*tests for each) between the effectiveness of inhibitions of tension, frequency, or amplitude after the addition of carbachol (1 µM), histamine (100 µM), 5-HT (100 µM), NKA (300 nM), PGE2 (10 µM), and ATII (100nM, *p* = NSD for all).

## 4 Discussion

The extent of influence that either extracellular or intracellular Ca^2+^ has on smooth muscle contraction varies throughout the body, promoting a specific interest towards identifying the prominent sources of Ca^2+^ influx across different organs. Ca^2+^ has a clear role in mediating the contractile activity of the bladder (Ikeda and Kanai, 2008), but the specific source of the Ca^2+^ in the urothelium and lamina propria tissue layer has never been investigated. Extracellular Ca^2+^ is of particular interest as it plays an essential role in many physiologic functions throughout the human body, and stimulates not only contraction, but also key underlying Ca^2+^ -dependent systems, which could be altered in bladder disorders (de Groat, 2004). As such, identifying the potential mechanisms involved in receptor-mediated contractions can support the development of new and novel pharmaceuticals, as well as develop a greater understanding of potential mechanisms underlying bladder dysfunction.

This study has identified prominent extracellular Ca^2+^ influences on muscarinic, histamine, 5-HT, neurokinin-A, prostaglandin E2 and angiotensin-II receptor mechanisms of action. Each of these receptor systems are G protein-coupled receptors of the G_q11_ class. Particular subtypes of interest have been previously identified as the M3 muscarinic (Moro et al., 2011), H1 histamine (Stromberga et al., 2019), 5-HT_2A_ serotonergic (Moro et al., 2016), neurokinin (Grundy et al., 2018), EP1 prostaglandin (Stromberga et al., 2020b), and AT_1_ angiotensin II (Lim et al., 2021) receptors. This broad presence of GPCRs in the U&LP constitutes the majority of surface receptors in the bladder, and many can be activated through neurotransmitters, hormones, and external stimuli that elicit a variety of cellular responses to stimulate downstream signalling activities (Mizuno and Itoh, 2009). Both inhibition of the L-type Ca^2+^ channels with nifedipine or inhibiting Ca^2+^ influx from extracellular fluids showed similar influences towards impairing GPCR contractions, inhibiting 20–50% of the contractile responses. These results are consistent with the finding by Heppner et al. (2011) where the inhibition of L-type voltage-gated Ca^2+^ channels reduced carbachol-induced contractions of human tissue by 74%, 18% in pig and 27% in mouse tissue. The additional finding that spontaneous phasic activity is not affected by the addition of nifedipine suggests that the overall conductance which generates the spontaneous contractions does not appear to have a relation with the L-type Ca^2+^ channels.

In this study, the response of each of the GPCRs to nifedipine has supported the presence of L-type voltage-gated Ca^2+^ channels in the U&LP of the urinary bladder. Lining the bladder lumen, the urothelium not only has an integral role in acting as the highly resistant physical barrier between urine and the underlying tissues, but also responds to stimuli and can transfer information to underlying cells (Birder and Andersson, 2013). Heppner et al. (2011) also observed the bladder U&LP of guinea pig tissue to exhibit dependence on Ca^2+^ influx through L-type Ca^2+^ channels, with spontaneous contractions significantly inhibited by nifedipine. However, clear evidence is lacking on the location of the L-type Ca^2+^ channels within these tissue layers. The lamina propria, a layer of highly innervated connective tissue located between the basement membrane of the urothelium and luminal surface of the detrusor, has demonstrated an essential role in signalling functions (Andersson and McCloskey, 2014; Heppner et al., 2017). In particular, its role in Ca^2+^ signalling may be of importance to the maintenance of normal bladder function and may present as a core site of extracellular Ca^2+^ influence. This could be through myofibroblasts, which have been identified to contribute to spontaneous activity through extracellular sources, and also express muscarinic and purinergic receptors that could assist in propagating signals to the urothelium (Heppner et al., 2011). Moreover, it has been suggested that the muscularis mucosae found within the underlying lamina propria of some species may also be impacted by the entry of extracellular Ca^2+^ (Heppner et al., 2011). In addition, interstitial cells of Cajal-like cells, located within the lamina propria layers, have also demonstrated firing activity of Ca^2+^ transients (Hashitani et al., 2004; Johnston et al., 2008), and there is some influence on the activity of pericytes surrounding blood vessels within the tissue (Mitsui and Hashitani, 2020).

The partial reduction (20–50%) in GPCR-mediated contractions of the U&LP by nifedipine may indicate additional sources of Ca^2+^ entry. This could include other voltage-gated channels such as T-type or P-type Ca^2+^ channels, previously identified in the urinary bladder (Deng et al., 2012). In addition, this partial reduction which was maintained in a Ca^2+^ -free environment suggests internal stores of Ca^2+^, such as from the sarcoplasmic reticulum, or other signalling pathways activated by G_q/11_ receptor proteins, may play a role in mediating contractile activity of the U&LP.

### 4.1 Clinical Relevance

Antimuscarinics and parasympathomimetics have shown success for the management of bladder contractile disorders, and currently sit as first-line pharmaceutical treatments for overactive bladder and underactive bladder, respectively. However, most patients cease treatment regimens due to lower than expected benefits and adverse side effects. Recent success has been found with combination therapies and there is increasing interest in the identification of alternative receptors systems that may be involved in contraction, and hence future targets for therapeutic treatments. This study’s identification of similarities between receptors mediating and modulating contraction in the urinary bladder may present future therapeutic targets or provide insights into mechanisms that may be dysfunctional in overactive or underactive bladder. Of particular interest was this study’s finding that histamine, neurokinin-A and angiotensin II are highly dependent on extracellular Ca^2+^, warranting further investigation into their clinical use for the management of bladder dysfunction.

### 4.2 Limitations and Future Direction

A limitation of this study was the use of single-dose applications of receptor agonists to examine changes in frequencies and amplitudes of phasic contractions over 30-min time periods. It should be noted that in detrusor studies of carbachol-induced bladder contractions, human tissue responded to inhibited extracellular Ca^2+^ to a greater extent than pig tissue (Wuest et al., 2007), however, this has not been demonstrated in urothelial and lamina propria studies. Future studies could investigate the potential role of ageing in influencing receptor responses to extracellular contractions, such as histamine (Stromberga et al., 2020a), as well as explore the influence of other GPCRs and the influence of Ca^2+^ on their responses.

## 5 Conclusion

This study identified a prominent role of extracellular Ca^2+^ for urinary bladder contractile activity. The responses obtained from muscarinic, histamine, 5-HT, neurokinin-A, prostaglandin E2 and angiotensin II receptor activation are highly sensitive to extracellular Ca^2+^, presenting a potential mechanism underlying bladder dysfunction.

## Data Availability

The raw data supporting the conclusion of this article will be made available by the authors, without undue reservation.
